# On a New Material and Process for Making Minute Anatomical Injections

**Published:** 1850-01

**Authors:** 


*On a new material and process for making Minute Anatomical Injections.* By Paul B. Goddard, M. D.—Having received recently from Europe some beautiful microscopic preparations, consisting of minute injections by Prof. Hyrtyl, Messrs. Hett, Dancer and Topping, I was stimulated to make an effort to obtain similar results, as they were, by far, finer than any which had been produced in this country. With the assistance of my friend Dr. Neill, demonstrator in the University of Pennsylvania, I made many experiments with variable results, but with such success as to lead to further investigation. At last I struck upon a plan which is uniformly productive of exquisitely beautiful results, and is moreover easy of application. For the purpose of making such an injection, the anatomist must provide himself with a small and good syringe; some vermilon *very finely* ground in oil; a glass stoppered bottle, and some sulphuric ether. The prepared vermillion paint must be put into the ground stoppered bottle, and about twenty or thirty times its bulk of sulphuric ether added; the stopper must then be put in its place and the whole well shaken. This forms the material of the injection. Let the anatomist now procure the organ to be injected (say a sheep's kidney, which is very difficult to inject in any other way, and forms an excellent criterion of success,) and fix his pipe in the artery, leaving the *vein open.* Having given his material a good shake, let him pour it into a cup and fill the syringe. Now, inject with a *slow, gradual,* and *moderate* pressure. At first the matter will return by the vein colored, but in a few moments this will case, and nothing will appear except the clear ether which will distill freely from the patulous vein. This must be watched, and when it ceases the injection is complete. The kidney is now to be placed in warm water of 120° Fahrenheit for a quarter of an hour, to drive off the ether, when it may be sliced and dried, or preserved in alcohol, Goadby's solution, or any other anti-septic fluid. For glands, as the kidney, liver, &c., it is better to dry and mount the sections in Canada balsam; but for membranous preparations, stomach, intestines, &c., the plan of mounting in a cell, filled with an anti-septic solution is preferable.—*Medical Examiner.*

